# Engineering thermoelectric and mechanical properties by nanoporosity in calcium cobaltate films from reactions of Ca(OH)_2_/Co_3_O_4_ multilayers†

**DOI:** 10.1039/d2na00278g

**Published:** 2022-07-04

**Authors:** Binbin Xin, Erik Ekström, Yueh-Ting Shih, Liping Huang, Jun Lu, Anna Elsukova, Yun Zhang, Wenkai Zhu, Theodorian Borca-Tasciuc, Ganpati Ramanath, Arnaud Le Febvrier, Biplab Paul, Per Eklund

**Affiliations:** Thin Film Physics Division, Department of Physics, Chemistry and Biology (IFM), Linköping University SE-58183 Linköping Sweden binbin.xin@liu.se per.eklund@liu.se; Department of Materials Science and Engineering, Rensselaer Polytechnic Institute Troy New York 12180 USA; Rensselaer Polytechnic Institute, Department of Mechanical, Aerospace, and Nuclear Engineering Troy NY 12180 USA

## Abstract

Controlling nanoporosity to favorably alter multiple properties in layered crystalline inorganic thin films is a challenge. Here, we demonstrate that the thermoelectric and mechanical properties of Ca_3_Co_4_O_9_ films can be engineered through nanoporosity control by annealing multiple Ca(OH)_2_/Co_3_O_4_ reactant bilayers with characteristic bilayer thicknesses (b_*t*_). Our results show that doubling b_*t*_, *e.g.*, from 12 to 26 nm, more than triples the average pore size from ∼120 nm to ∼400 nm and increases the pore fraction from 3% to 17.1%. The higher porosity film exhibits not only a 50% higher electrical conductivity of *σ* ∼ 90 S cm^−1^ and a high Seebeck coefficient of *α* ∼ 135 μV K^−1^, but also a thermal conductivity as low as *κ* ∼ 0.87 W m^−1^ K^−1^. The nanoporous Ca_3_Co_4_O_9_ films exhibit greater mechanical compliance and resilience to bending than the bulk. These results indicate that annealing reactant multilayers with controlled thicknesses is an attractive way to engineer nanoporosity and realize mechanically flexible oxide-based thermoelectric materials.

## Abstract 2

The higher porosity film with bending flexibility exhibits not only a 50% higher electrical conductivity of ∼90 S cm^−1^ and a high Seebeck coefficient of ∼135 μV K^−1^, but also a thermal conductivity as low as ∼1 W m^−1^ K^−1^.

## Graphical abstract

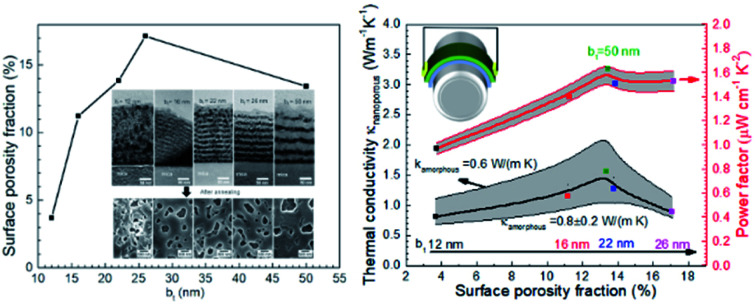

## 1. Introduction

The rapid development of autonomous, portable and wearable devices and sensors has sparked a great deal of interest in self-sustaining energy sources to replace batteries that are typically limited by shape constraints, periodic recharging and replacement.^1,2^ Harvesting electricity from heat using devices made from mechanically flexible thermoelectric materials is promising for such applications.^3–6^ Besides a high thermoelectric figure of merit ZT = *α*^2^*σT*/*κ* (*α* is the Seebeck coefficient, *σ* and *κ* the electrical and thermal conductivities, respectively, and *T* the absolute temperature^7–9^) for efficient energy conversion, properties such as high mechanical flexibility and toughness are key requirements.

Conducting polymer-based organic thermoelectric materials are mechanically flexible and exhibit ZT values as high as 0.42,^10–17^ but are unsuitable for higher than near-room-temperature applications. Purely inorganic thermoelectrics^18^ are usually brittle. Integrating nanograined films or nanocrystal assemblies of inorganic thermoelectrics, *e.g.*, Bi_2−*x*_Sb_*x*_Te_3_, on flexible substrates that can withstand moderately high temperatures addresses these challenges to some extent, and yield *α*^2^*σ* values up to 0.2 mW m^−1^ K^−2^ at ∼200 °C.^19,20^ Semiconductors with extraordinary metal-like ductility, *e.g.*, Ag_2_S, and Ag_2_Se also hold promise as free-standing thermoelectric materials that obviate flexible substrates.^21–23^

Oxide-based thermoelectric films on layered substrates such as mica offer greater stability at even higher temperatures and can become viable alternatives if high *α* and *σ*, are achieved together with high mechanical flexibility.^24,25^ Calcium cobaltate Ca_3_Co_4_O_9_ is an attractive p-type thermoelectric that exhibits inherently high *α* and high *σ* due to its layered crystal structure.^26–30^ The thermoelectric properties of layered cobaltates can be improved by nanostructuring, such as fabricating a high-quality single-phase,^31,32^ nanostructuring approaches,^33^ elemental doping,^34^ growing textured films with *c*-axis orientation.^35^ However, the thermal conductivity was normally increased with the improving electrical conductivity.^36,37^ Introducing nanoscale with dimensions mainly in the ranges of phonon mean free paths is a possible approach to reduce thermal conductivity without inhibiting electrical conductivity due to phonon mean free paths are typically significantly higher than electronic mean free paths. The textured nanograins and faceted nanopores not only offer additional means to lower the *κ*,^38,39^ but also alleviate the brittleness.^40,41^ Flexible porous nanograined Ca_3_Co_4_O_9_ films obtained by annealing CaO/CoO reactant multilayers exhibit power factors as high a 0.23 mW m^−1^ K^−2^ at room temperature.^40^ To further increase the porosity of nanoporous Ca_3_Co_4_O_9_ films retaining *α* and *σ*, it might be an effective way to further reduce *κ* and improve flexibility.

Here, we demonstrate that nanoporosity characteristics in Ca_3_Co_4_O_9_ films can be controlled by adjusting the reactant bilayer thickness in multilayer stacks. The porosities change from 11.2 to 17.1%. The nanoporous films show high *σ* with a narrow range from 80 to 95 S cm^−1^ and high *α* of ∼130 to ∼135 μV K^−1^, which are close to the values.^42–44^ The film with the highest porosity 17.1% has the lowest *κ* (∼0.87 W m^−1^ K^−1^). The nanoporous Ca_3_Co_4_O_9_ films exhibit greater mechanical compliance and resilience to bending than the bulk. Our findings showing pore engineering of layered-ceramics is attractive for realizing mechanically flexible thermoelectrics.

## 2. Experimental section

Nanoporous Ca_3_Co_4_O_9_ thin films were synthesized by annealing Ca(OH)_2_/Co_3_O_4_ multilayers on muscovite mica(00*l*) substrates. CaO/Co_3_O_4_ multilayers were deposited at 600 °C by reactive RF-magnetron sputtering from Ca and Co targets with a 2 mTorr plasma of a 0.5% O_2_/99.5% Ar gas mixture^40^. Ambient air-exposure of the CaO/Co_3_O_4_ multilayers for one month resulted in Ca(OH)_2_/Co_3_O_4_ multilayers *via* CaO hydration into Ca(OH)_2_.^45^ Subsequent annealing Ca(OH)_2_/Co_3_O_4_ multilayers at 700 °C for 2 hours in air led to nanoporous Ca_3_Co_4_O_9_ formation. We prepared five sets of multilayers with different Ca(OH)_2_/Co_3_O_4_ bilayer thicknesses, b_*t*_. The deposition times for CaO and Co_3_O_4_ were varied while keeping the total nominal multilayer thickness constant at ∼140 nm. The individual thicknesses of Ca(OH)_2_ and Co_3_O_4_ layers in each multilayer film were identical, *i.e.*, b_*t*_/2. Thus, altering the bilayer thickness in the 12 ≤ b_*t*_ ≤ 50 nm range is equivalent to varying the number of bilayers b_*n*_ in the 21 ≥ b_*n*_ ≥ 5 range.

Phase identification was carried out by X-ray diffractometry (XRD) using a PANalytical X'Pert PRO instrument with monochromatic Cu Kα radiation (*λ* = 1.5406 Å) and a Ni filter. X-ray reflectivity (XRR) measurements were carried out in a PANalytical Empyrean diffractometer equipped with a copper Cu Kα source with a hybrid mirror on the incidence beam path, a triple-axis Ge 220 analyzer on the diffracted beam path, and a PIXcel3D detector operated in open detection mode. The XRR data were fitted using the X'Pert reflectivity program.

Scanning electron microscopy (SEM) and energy dispersive X-ray (EDX) spectroscopy were carried out in a LEO Gemini 1550 Zeiss instrument operated at 10 kV to characterize film morphology and composition. The surface porosity fraction was determined by analyses of SEM micrographs using the Java version of image J software^46,47^. Most of the nanopores were hexagonal in shape. The average nanopore sizes were estimated from the nanopore area with an uncertainty of 20%, by assuming all the pores to be regular hexagons. The average nanopore size is equal to *L* + 2*L* cos 60° where *L* is hexagon side length. Transmission electron microscopy (TEM) was carried out in a FEI Tecnai G2 TF20 UT instrument operated at 200 kV on multilayer cross-sections prepared by face-to-face gluing of two sample pieces and mounting them on a Ti grid. The samples were mechanically polished down to 50 μm and ion-milled in a Gatan system with 2–5 kV Ar^+^ beams incident at 5°.

Electrical conductivity *σ* was determined from the sheet resistance measured with a four-point probe Jandel RM3000 station and the film thickness determined from cross-sectional TEM images. Seebeck coefficient *α* was determined from the slope of the temperature gradient–voltage characteristics measured in a homemade Seebeck measurement setup system equipped with two K-type thermocouples placed at the same position as two Cu electrodes, a Keithley 2001 multimeter, two Peltier elements acting as temperature controller, and two thermometers^45,48^.

Thermal conductivity of the annealed Ca_3_Co_4_O_9_ film-on-mica samples was determined by non-contact scanning thermal microscopy (SThM) described elsewhere^49–52^. This technique utilizes a Joule-heated 5 μm-diameter Wollaston wire probe, whose thermal resistance was measured in air at 100 nm above the sample surface based on temperature-induced changes in the electrical resistance of the probe and the dissipated Joule heating power. For each film, we measured thermal resistance at three different locations on the sample surface. Thermal conductivity was determined by fitting the thermal resistance data with a 3D finite element model (3DFEM) of the probe-to-sample surface heat transfer assuming isotropic thermal properties for the films and the substrate.

Mechanical flexibility was evaluated by estimating the elastic moduli of the films by surface Brillouin scattering (SBS) spectroscopy and measuring bending-induced relative changes in electrical resistance. SBS spectra were collected by using a JSR Scientific Instruments six-pass high-contrast Fabry–Perot interferometer equipped with a 532.18 nm Verdi V2 DPSS green laser probe. Surface Rayleigh and Sezawa wave velocities were obtained by using *V* = (*λ*_0_Δ*f*)/(2 sin *θ*_s_), where *λ*_0_ is the laser wavelength, Δ*f* the Brillouin frequency shift, and *θ*_s_ the scattering angle.^53^ For the bending tests, we measured the normalized electrical resistance change Δ*R*/*R*_0_ for different bending radii and cycles, where the initial resistance *R*_0_ includes contact resistances. A constant resistance during bending corresponds to Δ*R*/*R*_0_ = 0 and indicates good mechanical flexibility and retention of electrical properties.

## 3. Results and discussion

**Fig. 1:**
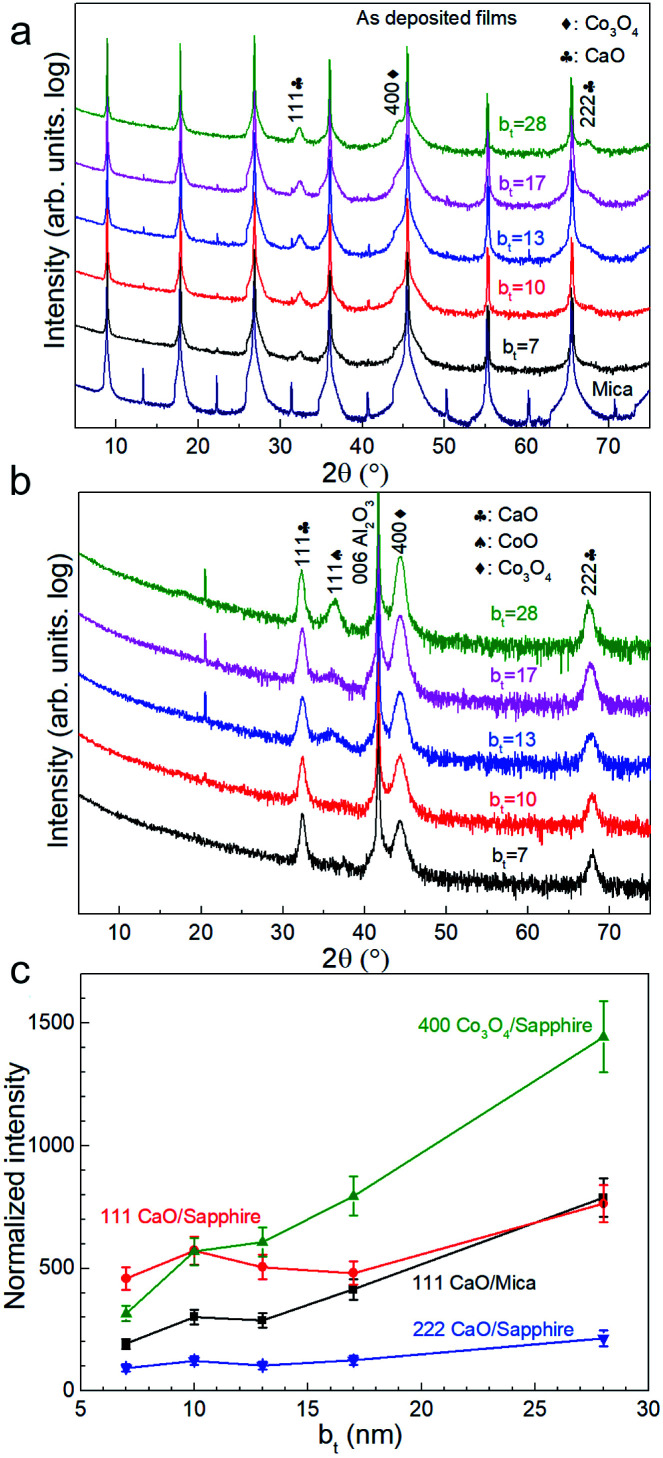
X-ray diffractograms from as-deposited CaO/Co_3_O_4_ multilayers with different bilayer thicknesses b_*t*_, on (a) mica (00*l*), and (b) sapphire (006) substrates. (c) CaO and Co_3_O_4_ peak intensities normalized with the intensity of the corresponding substrate peak, plotted as a function of b_*t*_.

X-ray diffractograms acquired immediately after deposition for all b_t_ values investigated show 111 and 222 Bragg reflections from CaO besides those from the mica substrate (Fig. 1a). The 400 diffraction peaks reflected from Co_3_O_4_ is overwhelmed by a stronger 00*l* reflection from the mica substrate (Fig. 1a) but is observable in diffractograms from as-deposited films on sapphire (Fig. 1b). Films on sapphire also show a weak 111 peak from CoO. The CaO and Co_3_O_4_ peaks intensities, normalized to that of the 004 mica substrate reflection and sapphire reflection, increase with increasing b_*t*_ (Fig. 1c).

**Fig. 2:**
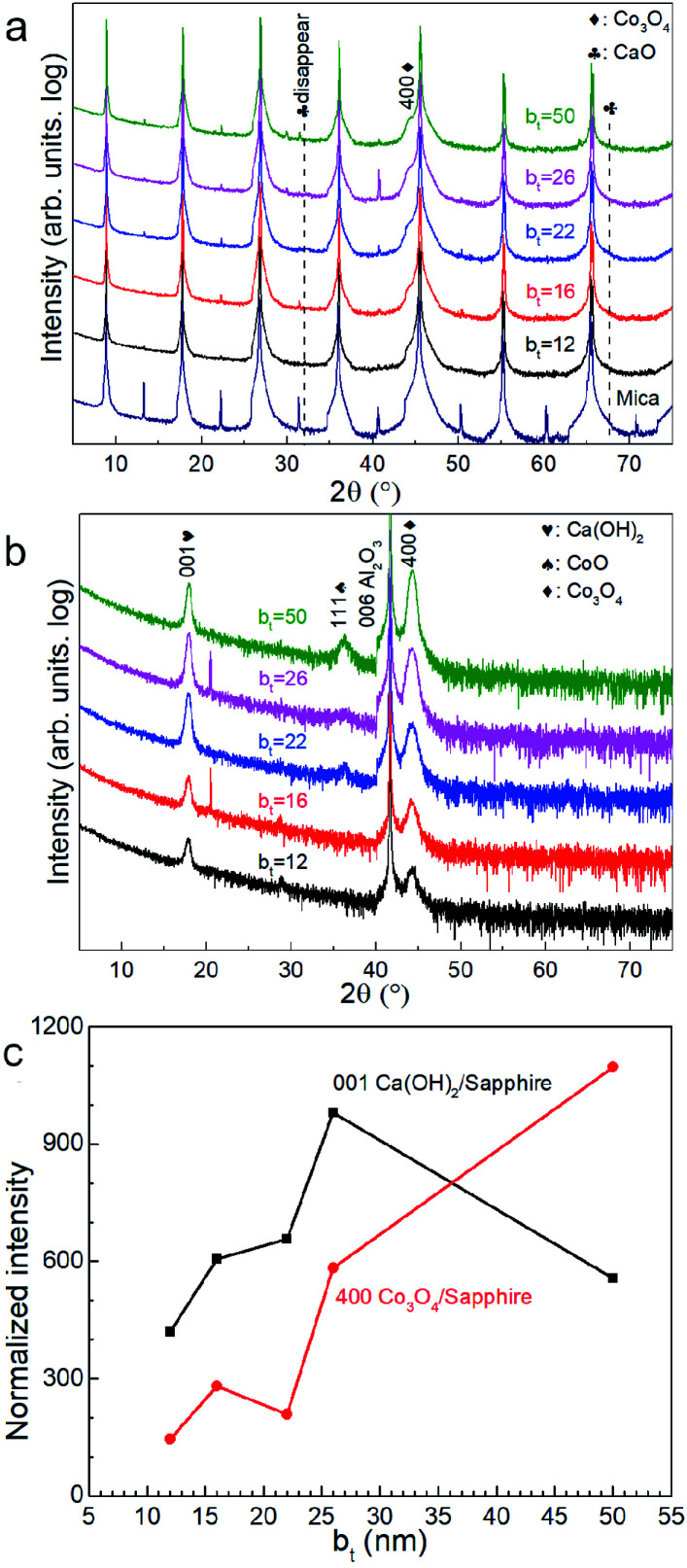
X-ray diffractograms from Ca(OH)_2_/Co_3_O_4_ multilayers formed by air-exposure of CaO/Co_3_O_4_ multilayers with different bilayer thicknesses between 12 ≤ b_*t*_ ≤ 50 nm on (a) mica (00*l*) and (b) sapphire (006) substrates. (c) Ca(OH)_2_ and Co_3_O_4_ peak intensities normalized to that of the 006 peak from the sapphire substrate, plotted as a function of b_*t*_.

Air-exposure of the as-deposited multilayers growing on mica and sapphire leads to the diminution and eventual disappearance of the CaO 111 and 222 peaks (see Fig. 2a and b). Multilayers on sapphire also show a similar behaviour besides revealing a concomitant emergence of the Ca(OH)_2_ 001 peak. This peak is not observable in diffractograms from films on mica due to substrate peak overlap. These results are indicative of the conversion of CaO to Ca(OH)_2_ through ambient moisture uptake.^45^ We note that the intensities of the Ca(OH)_2_ and Co_3_O_4_ peaks increase with increasing b_t_, the intensity of Ca(OH)_2_ is not the highest when the b_t_ is 50 nm (Fig. 2c).

**Fig. 3:**
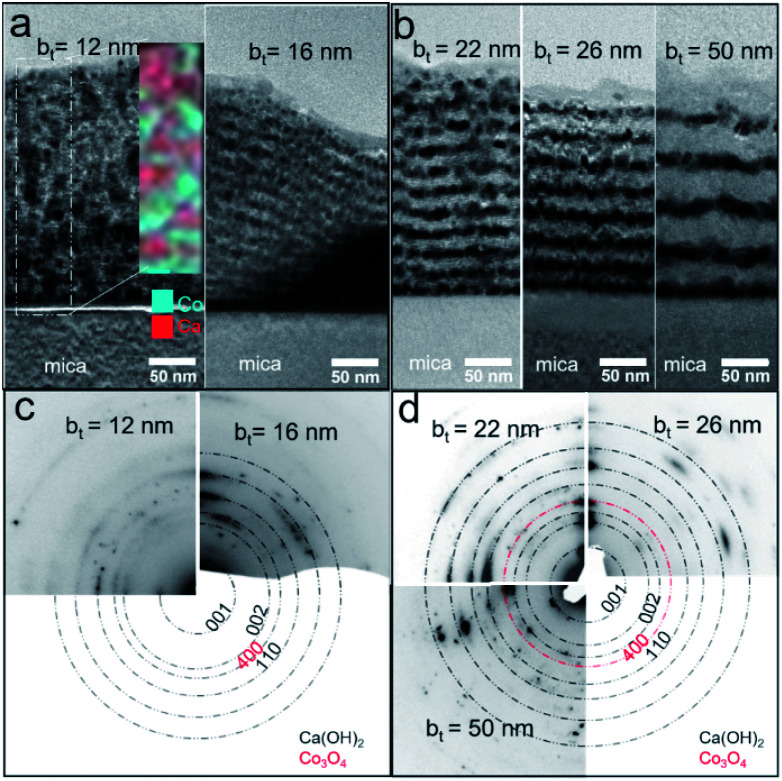
(a and b) Cross-sectional bright-field TEM images and (c and d) selected-area electron diffraction (SAED) patterns from Ca(OH)_2_/Co_3_O_4_ multilayers with different bilayer thicknesses b_*t*_.

Air-exposed CaO/Co_3_O_4_ multilayers specified by low b_*t*_ (*e.g.*, ∼12 nm) consist of equiaxed grains of Ca(OH)_2_ and Co_3_O_4_ without any discernible interfaces (Fig. 3a). EDX spectral maps of Ca and Co indicate that Ca(OH)_2_ and Co_3_O_4_ are interspersed across individual layers (Fig. 3a inset). This result is consistent with Co_3_O_4_ layers comprised of discontinuous grains that facilitate moisture intake and transport, as suggested in our recent work.^45^ Air-exposed multilayers with b_*t*_ = 16 nm exhibit more distinct Ca(OH)_2_/Co_3_O_4_ interfaces (Fig. 3b). The Ca-containing layers that appear brighter due to a higher Z-contrast are about 81% thicker than the Co_3_O_4_ layers, consistent with a 95.2% unit cell volume expansion caused by the CaO → Ca(OH)_2_ conversion during air-exposure. For b_*t*_ > 16 nm, TEM images show distinct Co- and Ca-containing layers, but with a greater interface roughness that increases with b_*t*_. Such interface roughening is attributable to the higher volume Ca(OH)_2_ grains encroaching into the nearly unchanged adjacent Co_3_O_4_ layers. For b_*t*_ = 50 nm, the thickness of Ca(OH)_2_ layer in top larger than that in bottle near mica, which may be due to the low hydration reactions by preventing of thicker Co_3_O_4_ layers and lead to the low intensity of Ca(OH)_2_ 001 in Fig. 2c. SAED patterns indicate increased in-plane grain texturing with increasing b_*t*_ (see Fig. 3c and d). EDX spectral maps from air-exposed films with higher b_*t*_ show distinct Co-containing layers, in contrast to the uniform distribution of Ca, indicating Ca diffusion (see ESI Fig. S1†). Our results indicate that the bilayer thickness needs to be greater than a critical value of b_*t*_ > 12 nm to form layers structure with sharp interfaces.

Otherwise, EDS maps from multilayers with b_*t*_ = 16 nm show clear Co layers and almost uniform distribution Ca elements in multilayers (see ESI Fig. S1a†).

**Fig. 4:**
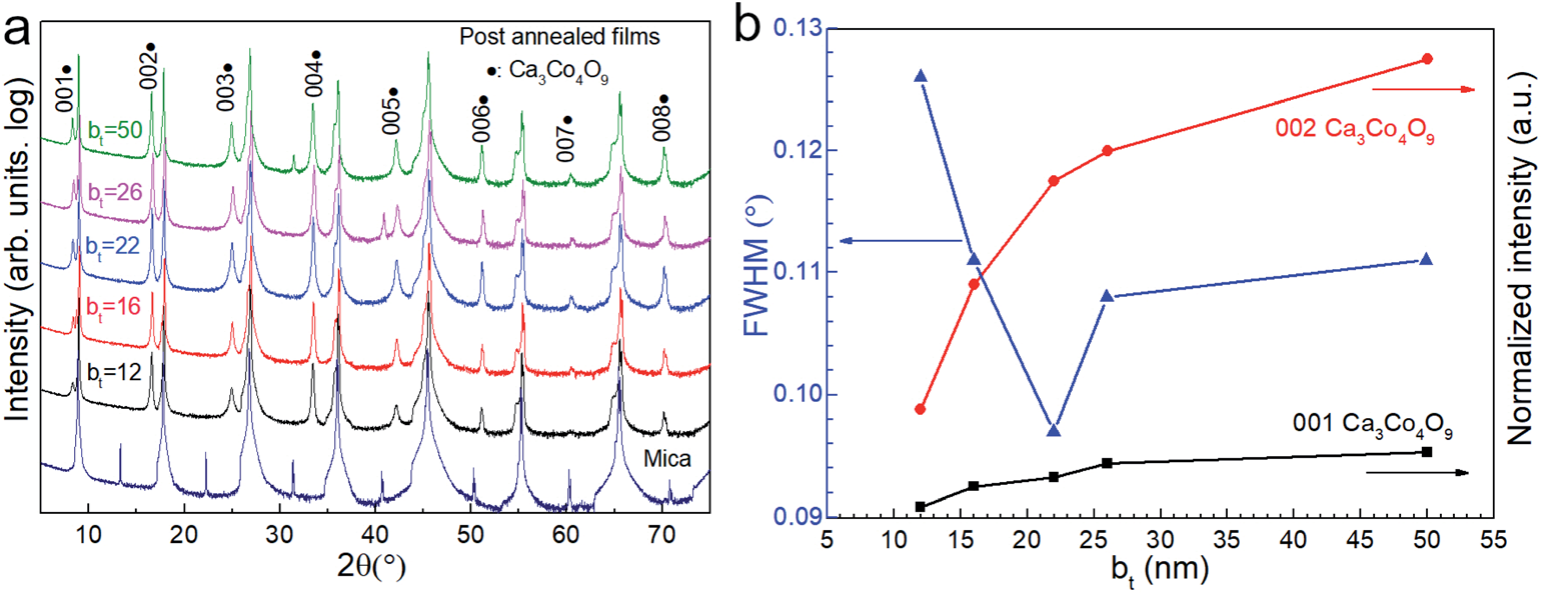
(a) X-ray diffractograms showing Ca_3_Co_4_O_9_ formation on mica (00*l*) substrate by annealing Ca(OH)_2_/Co_3_O_4_ multilayers with different bilayer thicknesses in the 12 ≤ b_*t*_ ≤ 50 nm range. (b) Ca_3_Co_4_O_9_ 001 and 002 peak intensities normalized with the intensity of the corresponding substrate peaks, plotted as a function of b_*t*_.

X-ray diffractograms show that annealing the Ca(OH)_2_/Co_3_O_4_ multilayers leads to Ca_3_Co_4_O_9_ formation through a reaction between the Co_3_O_4_ and Ca(OH)_2_ layers (Fig. 4a). The exclusive presence of multiple 00*l* peak reflections of Ca_3_Co_4_O_9_ indicate textured basal planes oriented parallel to the substrate surface. The Ca_3_Co_4_O_9_ grain size is largest for films specified by b_*t*_ = 22 nm, as indicated by the narrowest width of the Ca_3_Co_4_O_9_ 002 reflection.

**Fig. 5:**
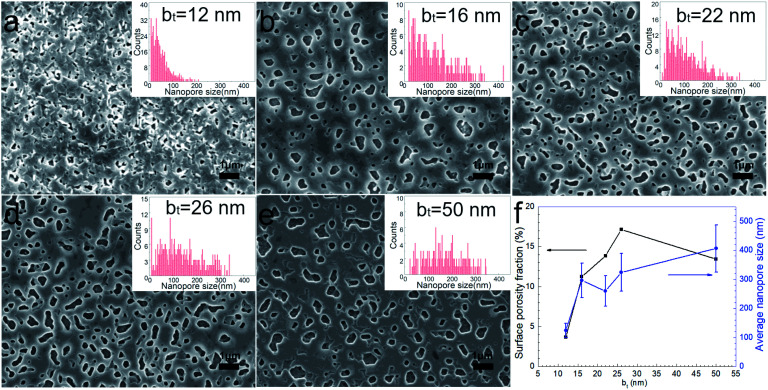
(a–e) SEM images from annealed Ca(OH)_2_/Co_3_O_4_ multilayers with 12 ≤ b_*t*_ ≤ 50 nm grown on mica (00l) substrates. (f) Surface porosity fraction determined from SEM image analyses.

Surfaces of the annealed films exhibit faceted intergranular nanopores (see Fig. 5). Films with the smallest b_*t*_ in our studies reveal a relatively rough surface, probably due to the edge-on orientation of some platelet-shaped grains on the surface (see Fig. 5a). In contrast, annealed films with b_*t*_ ≥ 16 nm exhibit smoother surfaces, with faceted nanopores between hexagonal terraces and plate-shaped grains (Fig. 5b–e). The average pore size increases monotonically from around 120 to 400 nm with increasing b_*t*_ from 12 nm to 50 nm (see Fig. 5f). The porosity fraction increases with b_*t*_ from 3.7% to 17.1% for 12 ≤ b_*t*_ ≤ 26 nm but drops to 13.4% for b_*t*_ = 50 nm (Fig. 5f).

**Fig. 6:**
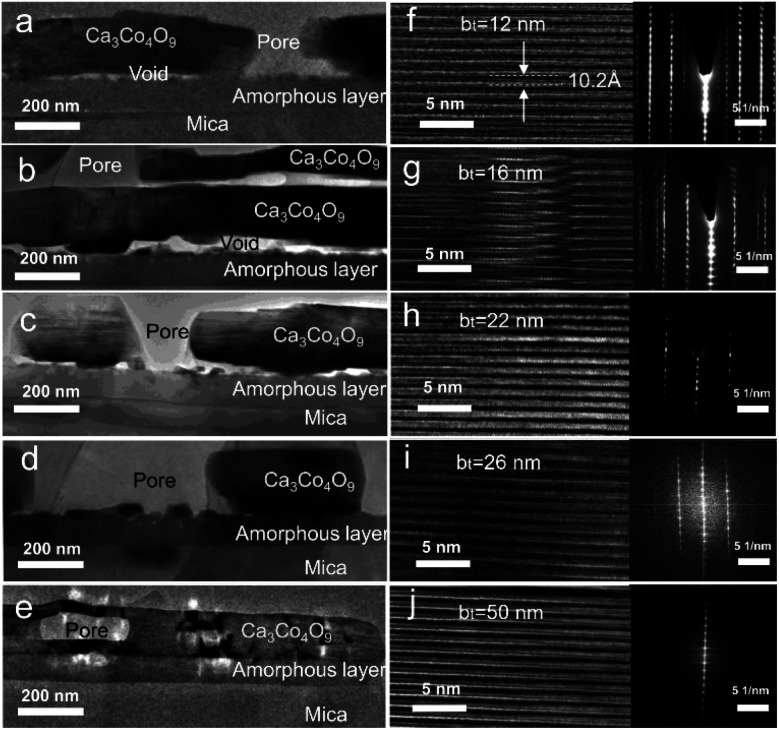
(a–e) Cross-sectional bright-field TEM images from Ca_3_Co_4_O_9_ films obtained by annealing Ca(OH)_2_/Co_3_O_4_ multilayers with 12 ≤ b_*t*_ ≤ 50 nm created from CaO/Co_3_O_4_ multilayers. (f–j) The corresponding high-resolution TEM images and SAED patterns capturing the layered atomic structure of Ca_3_Co_4_O_9_.

Cross-sectional TEM micrographs of films obtained by annealing Ca(OH)_2_/Co_3_O_4_ multilayers with 12 ≤ b_*t*_ ≤ 50 nm created from CaO/Co_3_O_4_ multilayers (Fig. 6a–e) reveal a polycrystalline Ca_3_Co_4_O_9_ layer separated from the substrate by an amorphous glass layer.^40^ Lattice images for the Ca_3_Co_4_O_9_ layer and SAED patterns (Fig. 6f–j) confirm that the (001) basal planes are oriented parallel to the film surface, corroborating our XRD and SEM results. The in-plane/out-of-plane aspect ratio of the pores is about four for the film with b_*t*_ = 50 nm (Fig. 6e). This observation is consistent with the SEM results and our recent work^45^ indicating that oriented nanopore formation arises from basal plane removal driven by local densification of textured Ca_3_Co_4_O_9_. For all b_*t*_ values except b_*t*_ = 50 nm, we observe nanopores spanning across the Ca_3_Co_4_O_9_ layers and microporous gaps at the Ca_3_Co_4_O_9_ – amorphous layer interface and interlayer nanoporosity (see Fig. 6a–e). No microporous gaps or interlayer porosity are discernible in the amorphous layers.

**Fig. 7:**
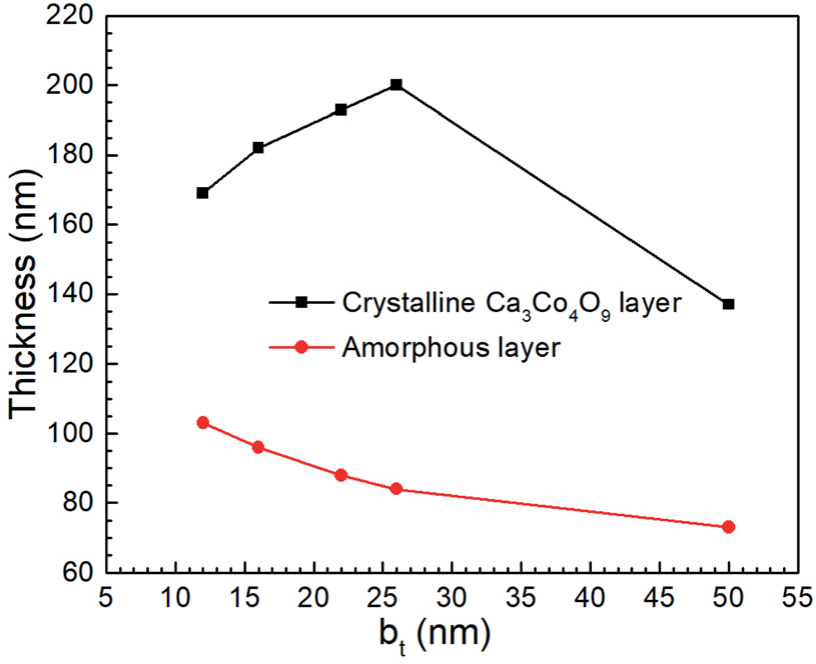
The thicknesses of crystalline Ca_3_Co_4_O_9_ and amorphous layers in porous films obtained by annealing Ca(OH)_2_/Co_3_O_4_ multilayers created from CaO/Co_3_O_4_ multilayers with 12 ≤ b_*t*_ ≤ 50 nm.

TEM micrographs reveal that for all our films, the Ca_3_Co_4_O_9_ layer thickness is around twice the amorphous layer thickness (Fig. 7). Increasing the b_*t*_ from 12 nm to 26 nm increased the Ca_3_Co_4_O_9_ layer thickness from 170 ± 10 nm to 193 ± 10 nm but decreased the amorphous layer thickness from 103 nm to 73 nm. EDX analyses of the amorphous layer revealed O, Al, Si and Ca (ESI Fig. S2†), but no traces of Co above the EDX detection limit. These results suggest that the amorphous layer is formed due to preferential Ca diffusion and incorporation into the mica substrate. This inference is supported by the inverse correlation between amorphous layer thickness and b_*t*_ and the higher Ca/Co ratio of 55 : 45 in multilayer than that in the Ca_3_Co_4_O_9_ layer. The anomalously low Ca_3_Co_4_O_9_ layer thickness of 159 ± 10 nm for b_*t*_ = 50 nm is likely an outlier due to a very low surface porosity fraction of 13.4% and no microporous gaps at the Ca_3_Co_4_O_9_ – amorphous layer interface and needs further study.

### Thermoelectric properties

**Fig. 8:**
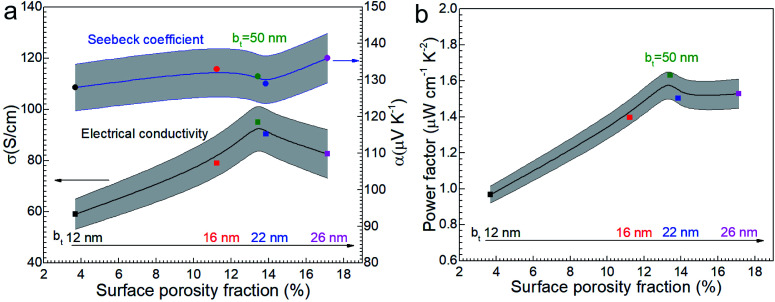
(a) The electrical conductivity *σ* and the Seebeck coefficient *α* and (b) power factor *α*^2^*σ* of nanoporous Ca_3_Co_4_O_9_ films as function of surface porosity fraction at room temperature.

Considering that amorphous materials normally have lower electrical conductivity *σ* than corresponding crystalline materials and the amorphous layer has a low thermal conductivity *κ*, the amorphous oxide layer is assumed as an insulator. The electrical conductivity *σ* of Ca_3_Co_4_O_9_ films slightly increases with b_*t*_ (Fig. 8a) and peaks around 13% porosity (Fig. 8b). For example, *σ* ∼ 90 S cm^−1^ for the film with higher porosity (13.8%) is 50% higher than for the film with lowest porosity (3.7%). But *σ* for the film with different porosities from 11.2 to 17.1% shows a narrow range from 80 to 95 S cm^−1^. The reason may be that the rough morphology and lowest quality layered structure of annealed film with b_*t*_ = 12 nm cause lower carrier mobility and the high-quality layered structure of the annealed films with b_*t*_ ≥ 16 nm led to high carrier mobility as shown in Fig. 4 and the size range of nanopores mainly from 120 nm to 400 nm has a slight effect for the electrical conductivity.

The Seebeck coefficient *α* increases sightly with nanoporosity fraction, *e.g.*, from *α* = 128 μV K^−1^ observed for porosity = 3.7% to *α* = 136 μV K^−1^ for porosity = 17.1%. The power factor increases from 0.96 to 1.53 μW cm^−1^ K^−2^ with increasing porosity (Fig. 8b). The highest power factor is *α*^2^*σ* = 1.63 μW cm^−1^ K^−2^ for the film with b_*t*_ = 50 nm with highest *σ*.

**Fig. 9:**
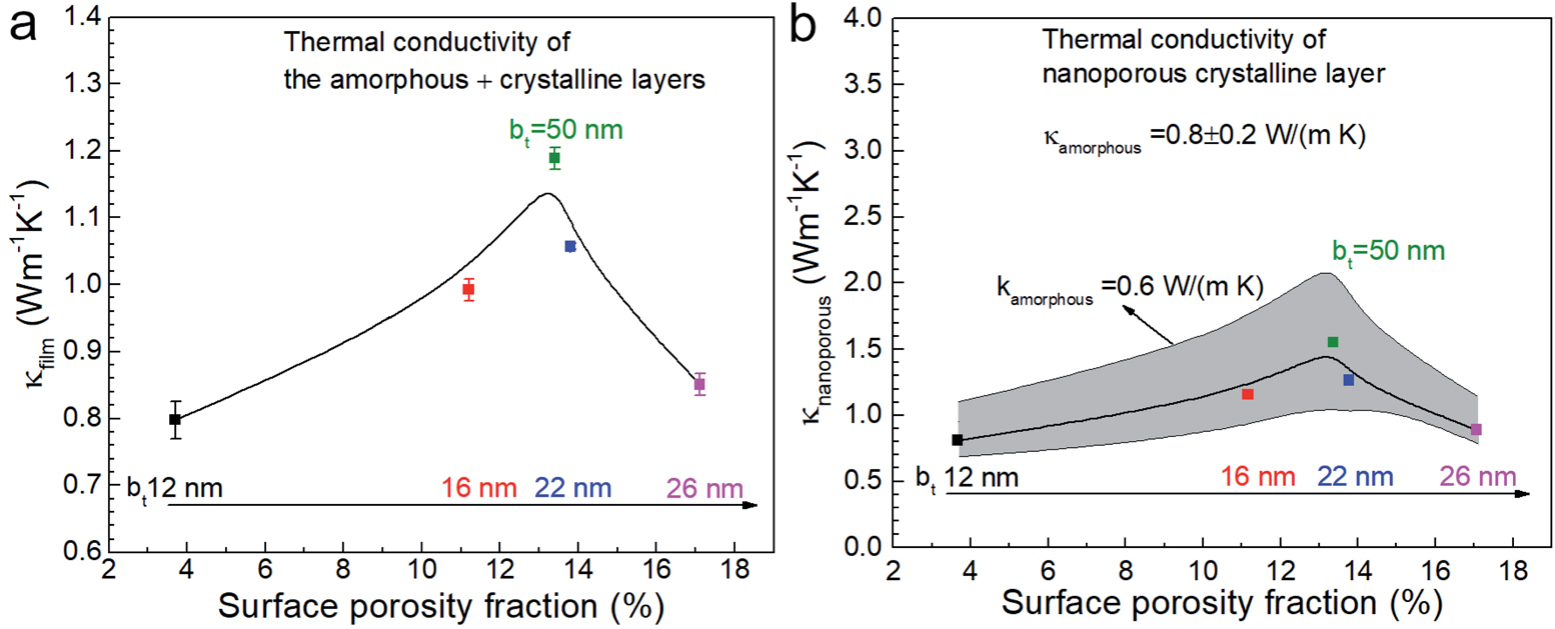
(a) Effective thermal conductivity *κ* film of Ca_3_Co_4_O_9_ films as a function of surface porosity fraction, and (b) thermal conductivity of the nanoporous crystalline Ca_3_Co_4_O_9_ layer *κ*_nanoporous_, assuming *κ*_amorphous_ = 0.8 W m^−1^ K^−1^ with a ±0.2 W m^−1^ K^−1^ uncertainty captured by error bars.

We used 3DFEM model fitting^49^ to determine the effective thermal conductivity *κ*_film_ of the Ca_3_Co_4_O_9_ films (see Fig. 9) by using a mica substrate thermal conductivity value of *κ*_sub_ = 0.421 W m^−1^ K^−1^, determined by SThM probe measurements. Since *κ*_film_ depends on the thermal conductivities of the amorphous (*κ*_amorphous_) and nanoporous crystalline (*κ*_nanoporous_) layers, both of which are unknown, we computed *κ*_nanoporous_ by using a one-dimensional cross-plane thermal resistance network model for the crystalline-amorphous bilayer described by 
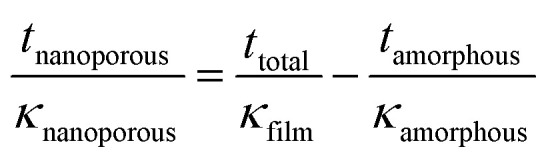
. We selected three possible *κ*_amorphous_ values of 0.6, 0.8, and 1 (0.8 ± 0.2) W m^−1^ K^−1^, which are comparable to the thermal conductivity of amorphous silica.^54^ We assumed *κ*_amorphous_ to be identical in all the five samples with 50 ≥ b_*t*_ ≥ 12 nm and used the measured amorphous layer thickness for each sample, *i.e.*, 73 nm ≤ *t*_amorphous_ ≤ 103 nm. This model is apt because both the amorphous and the nanoporous regions are much thinner than the ∼5 μm probe-sample heat transfer radius and have low values of fitted thermal conductivities.

Plotting the effective *κ*_film_ and the values extracted for *κ*_nanoporous_ of the nanoporous layer as a function of porosity (see Fig. 9a and b) reveal peaks in both *κ*_film_ and *κ*_nanoporous_ around 13% porosity, at which *σ* also peaks, as shown earlier. We note that decreasing assumed *κ*_amorphous_ yields a higher *κ*_nanoporous_. Both the *κ*_film_ and *σ* are low for the lowest porosity film due to the relatively higher surface roughness compared to the other films. The film with the highest porosity 17.1% (*i.e.*, with b_*t*_ = 26 nm) has the lowest *κ* (∼0.87 W m^−1^ K^−1^) in annealed films with b_*t*_ ≥ 16 nm. The annealed film with b_*t*_ = 12 nm shows low *κ* (∼0.8 W m^−1^ K^−1^) due to the rough morphology.

### Mechanical flexibility

**Fig. 10:**
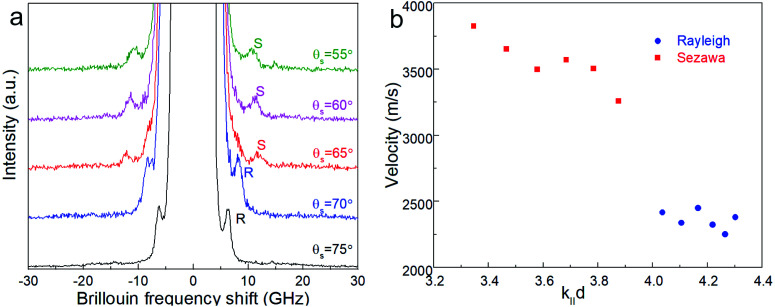
(a) Representative Brillouin spectra from a ∼185 nm-thick nanoporous Ca_3_Co_4_O_9_ film (corresponding to b_*t*_ = 26 nm) on sapphire for different scattering angles *θ*_s_ showing Rayleigh (R) and Sezawa (S) peaks. (b) Surface Rayleigh and Sezawa velocities plotted a of *k*‖*d*, where *k*‖ = (4π sin *θ*_s_)/*λ*_0_ is the surface acoustic wave vector, and *d* the film thickness.

Surface Brillouin scattering (SBS) spectra from Ca_3_Co_4_O_9_ films (Fig. 10a) reveal stiffness values that are significantly lower than that of bulk Ca_3_Co_4_O_9_. Rayleigh peaks are seen around ± 8.5 GHz at scattering angles *θ*_s_ ≥ 70°. At lower scattering angles, the Rayleigh peaks merge into the central elastic peak, and we observe Sezawa peaks around ±11.5 GHz. Sezawa peaks are typical of soft films on hard substrates,^55^ as is our case for Ca_3_Co_4_O_9_ on sapphire. The invariance of the surface Rayleigh velocity with *k*‖*d* (Fig. 10b) within experimental uncertainties is indicative of the elastic properties of the Ca_3_Co_4_O_9_ film with negligible influence of the sapphire substrate. In contrast, the Sezawa velocity increases substantially with decreasing *k*‖*d* due to the hard sapphire substrate. We calculated the shear modulus *G* of the nanoporous Ca_3_Co_4_O_9_ film from 
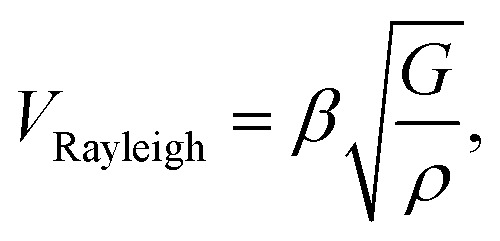
 where *ρ* is the density and *β* = 0.94.^56,57^ Assuming isotropy, invoking *E* = 2*G*(1 + *ν*), using the bulk Ca_3_Co_4_O_9_ Poisson's ratio *ν* = 0.31,^58^ and a density *ρ* ∼ 3.0 g cm^−3^ for the nanoporous Ca_3_Co_4_O_9_ film determined from X-ray reflectivity measurements (ESI Fig. S3†), we get Young's and shear moduli values of *G* = 18.92 ± 1.14 GP, and *E* = 49.62 ± 2.99 GPa, respectively. Both these values for nanoporous Ca_3_Co_4_O_9_ film are 52.7% lower than the predicted values of *G* = 39.98 GPa and *E* = 104.86 for bulk Ca_3_Co_4_O_9_.^58^ The 21% lower density of the nanoporous Ca_3_Co_4_O_9_ film compared to bulk Ca_3_Co_4_O_9_ (3.8 g cm^−3^) partially explains the lower moduli. We propose that the low atomic bond density near the nanopore walls in the film also contribute to the increased mechanical compliance.

**Fig. 11:**
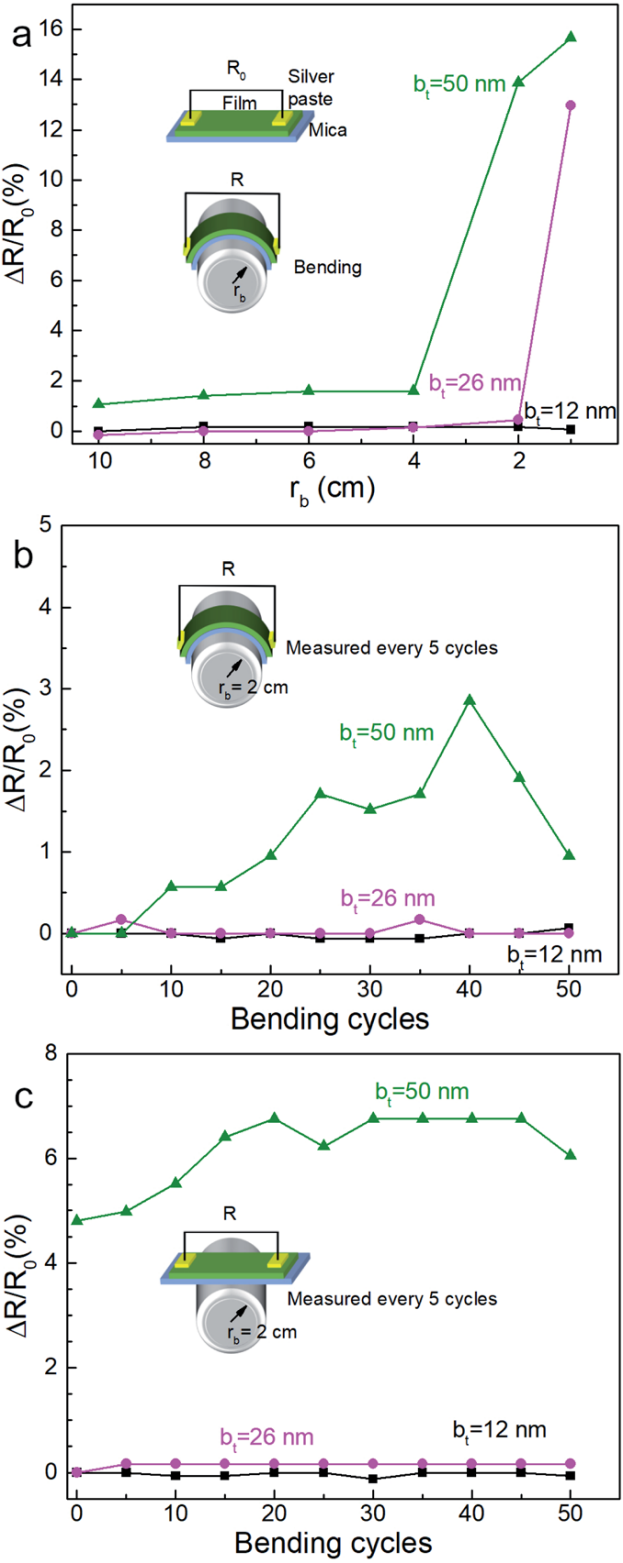
The normalized resistance changing Δ*R*/*R*_0_ of the Ca_3_Co_4_O_9_ films on mica (00*l*) substrates obtained by reactions of multiple stacks of Ca(OH)_2_/Co_3_O_4_ bilayers with 12 ≤ b_*t*_ ≤ 50 nm as a function of (a) bending radius *r*_b_ and (b) the number of bending cycles at a bending radius of 2 cm, and (c) after restoration to non-bent geometry.

Mechanical bending of the film-substrate composite showed a high retention of the electrical properties, consistent with high mechanical compliance indicated by the SBS results. Two-point resistance of the film measured as a function of bending radius *r*_b_ (see Fig. 11a) showed negligible changes in the normalized resistance (Δ*R*/*R*_0_ ∼ 0) for *r*_b_ ≥ 4 cm, where *R*_0_ is the initial resistance for each film, indicating good mechanical flexibility and low electromechanical coupling. Films synthesized from multilayers specified by b_*t*_ = 12 nm and b_*t*_ = 26 nm show a greater resilience to bending and bend cycling than the film corresponding to b_*t*_ = 50 nm (Fig. 11b and c). While further studies are needed to understand correlations between nanoporosity, bend cycling, and mechanical compliance, our results clearly indicate that the nanoporous films are mechanically flexible and have a low electromechanical coupling for a large range of b_*t*_ and bending.

## Conclusions

We have synthesized Ca_3_Co_4_O_9_ films with different porosities by annealing multilayers of calcium and cobalt oxide bilayers specified by Ca(OH)_2_/Co_3_O_4_ bilayer thicknesses b_*t*_. Increasing b_*t*_ increases the nanoporosity fraction and average nanopore size in the Ca_3_Co_4_O_9_ film formed during annealing. The higher porosity films exhibit a 50% higher electrical conductivity as well as a high Seebeck coefficient, together with a low thermal conductivity. The nanoporous Ca_3_Co_4_O_9_ films show a higher mechanical compliance than the bulk Ca_3_Co_4_O_9_ and are resilient to mechanical bending and bend cycling. These results indicate that engineering nanoporosity in layered oxides through reactions of multilayer stacks of component oxides could be attractive for achieving mechanically-flexible high-figure-of-merit thermoelectric nanomaterials for emergent applications.

## Author contributions

Binbin Xin: performed experiments, data collection and analysis, writing – original draft, and writing – review & editing; Erik Ekström: magnetron sputtering; Jun Lu and Anna Elsukova: TEM data; Yueh-Ting Shih and Liping Huang: surface Brillouin scattering (SBS) spectroscopy; Yun Zhang, Wenkai Zhu, and Theodorian Borca-Tasciuc: thermal conductivity; Biplab Paul and Per Eklund: experiment design and supervision; Erik Ekström, Yueh-Ting Shih, Liping Huang, Jun Lu, Anna Elsukova, Yun Zhang, Wenkai Zhu, Theodorian Borca-Tasciuc, Ganpati Ramanath, Arnaud Le Febvrier, Biplab Paul and Per Eklund: writing – review & editing.

## Conflicts of interest

The authors declare no conflicts of interest.

## Supplementary Material

NA-004-D2NA00278G-s001
