# Supplementary material for: Engineering thermoelectric and mechanical properties by nanoporosity in calcium cobaltate films from reactions of Ca(OH)2/Co3O4 multilayers
Source: Nanoscale Adv. 2022 Jul 4;4(16):3353–61. doi: 10.1039/d2na00278g (PMC9416876; doi:10.1039/d2na00278g)
Supplement: NA-004-D2NA00278G-s001 [file NA-004-D2NA00278G-s001.pdf]

Electronic Supplementary Material (ESI) for Nanoscale Advances.
This journal is © The Royal Society of Chemistry 2022

Supplementary information

Engineering thermoelectric and mechanical properties by nanoporosity in calcium cobaltate films from reactions of $Ca(OH)_2/Co_3O_4$ multilayers

Binbin Xin[a], Erik Ekström[a], Yueh-Ting Shih[b], Liping Huang[b], Jun Lu[a], Anna Elsukova[a], Yun Zhang[c], Wenkai Zhu[c], Theodorian Borca-Tasciuc[c], Ganpati Ramanath[a,b], Arnaud Le Febvrier[a], Biplab Paul[a] and Per Eklund[a*]*

[a]Thin Film Physics Division, Department of Physics, Chemistry and Biology (IFM), Linköping University, SE-58183 Linköping, Sweden

[b]Department of Materials Science and Engineering, Rensselaer Polytechnic Institute, Troy, New York 12180, USA

[c]Rensselaer Polytechnic Institute, Department of Mechanical, Aerospace, and Nuclear Engineering, Troy, NY 12180, USA.

*E-mail: binbin.xin@liu.se; per.eklund@liu.se

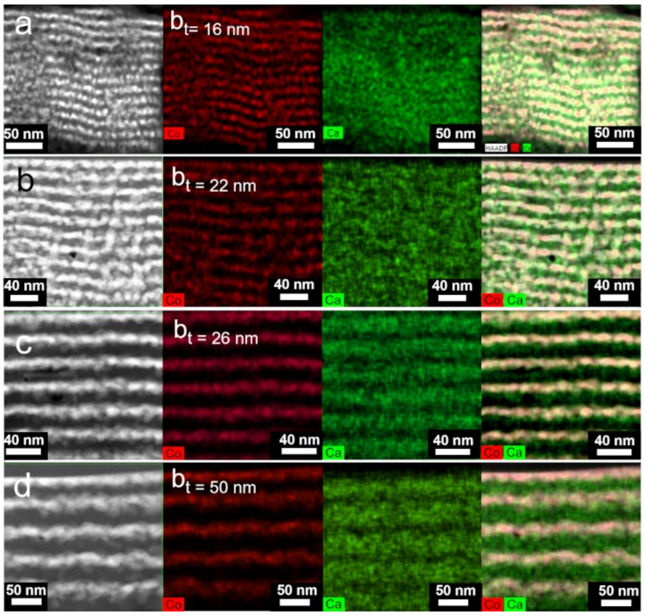

Figure S1. STEM image and EDX spectral maps of Co and Ca from as-deposited CaO/Co_3O_4 multilayer films with different $Ca(OH)_2/Co_3O_4$ bilayer thickness b_t formed after air-exposure for one month: (a) b_t= 15 nm, (b) b_t= 22 nm, (c) b_t= 26 nm, and (d) b_t= 50 nm.

STEM images indicate increasing interface roughness with increasing of b_t. The EDX spectral maps for b_t = 16 nm and 22 nm indicate distinct alternating Co-containing layers corresponding to Co_3O_4. In contrast, the Ca is distributed uniformly across the multilayers (Fig. S1a and 1b), indicating Ca diffusion. EDX spectral maps for higher b_t show distinct layers of Co and Ca (Fig. S1c and 1d), but with a greater interface waviness.

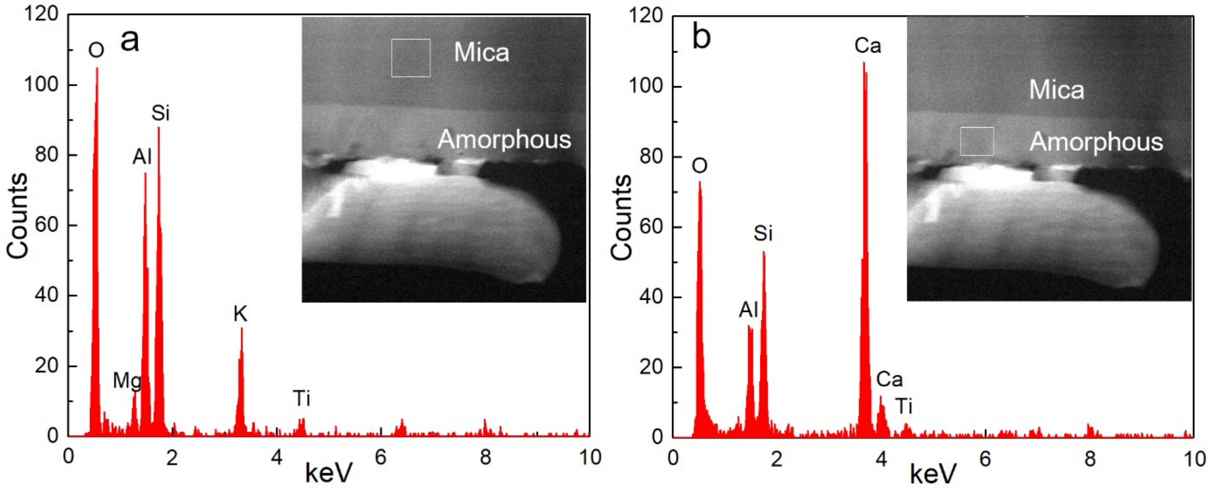

Figure S2. EDX spectra from (a) the mica substrate and (b) the interfacial amorphous layer formed during $Ca_3Co_4O_9$ thin film synthesis by annealing multilayers of $Ca(OH)_2/Co_3O_4$ specified by characteristic $Ca(OH)_2/Co_3O_4$ bilayer thickness b_t= 26 nm. Insets show the STEM images.

EDX spectra from mica shows O, Mg, Al, Si, and K (Fig. S2a). EDX spectra from the amorphous glass layer shows O, Al, Si, and Ca (Fig. S2b), suggesting that the interfacial amorphous layer is formed by the diffusion of Ca into, and reaction with, the mica substrate.

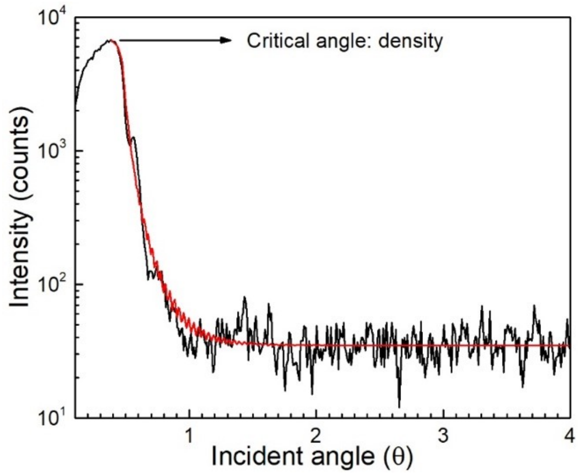

Figre S3. X-ray reflectivity (XRR) of $Ca_3Co_4O_9$ films on sapphire as a function of incidence angle.

Simulations (red) overlaid on the measured data revealed the film density (relate to the total external reflection angle) to be ~3 g cm^{-3}.
